# Recent advances of drug delivery nanocarriers in osteosarcoma treatment

**DOI:** 10.7150/jca.36588

**Published:** 2020-01-01

**Authors:** Shang-Yu Wang, Hong-Zhi Hu, Xiang-Cheng Qing, Zhi-Cai Zhang, Zeng-Wu Shao

**Affiliations:** Department of Orthopedics, Union Hospital, Tongji Medical College, Huazhong University of Science and Technology, Wuhan 430022, China

**Keywords:** Drug delivery, nanocarriers, osteosarcoma, stimuli-response, tumor-targeted

## Abstract

Osteosarcoma is the most common primary malignant bone tumor mainly occurred in children and adolescence, and chemotherapy is limited for the side effects and development of drug resistance. Advances in nanotechnology and knowledge of cancer biology have led to significant improvements in developing tumor-targeted drug delivery nanocarriers, and some have even entered clinically application. Delivery of chemotherapeutic agents by functionalized smart nanocarriers could protect the drugs from rapid clearance, prolong the circulating time, and increase the drug concentration at tumor sites, thus enhancing the therapeutic efficacy and reducing side effects. Various drug delivery nanocarriers have been designed and tested for osteosarcoma treatment, but most of them are still at experimental stage, and more further studies are needed before clinical application. In this present review, we briefly describe the types of commonly used nanocarriers in osteosarcoma treatment, and discuss the strategies for osteosarcoma-targeted delivery and controlled release of drugs. The application of nanoparticles in the management of metastatic osteosarcoma is also briefly discussed. The purpose of this article is to present an overview of recent progress of nanoscale drug delivery platforms in osteosarcoma, and inspire new ideas to develop more effective therapeutic options.

## Introduction

Chemotherapy is an important approach in cancer therapy. Effective treatment of cancers needs accurate delivery of an enough intracellular dose of chemo-drugs to kill the cancer cells 1. And chemotherapy for cancer is a delicate balance between response and toxicity, while low-dosing fails to obtain effective effects, over-dosing leads to excessive systemic toxicity 2. Furthermore, drug distribution efficiency from plasma to tumors is affected by some physiologic parameters, such as competitive drug uptake by liver, excretion of small molecule drugs by urine, drug inactivation by binding to proteins, and low stability of drug in fluids 3. Therefore, nanoscale drug delivery systems have been widely studied in recent years for tumor-targeted drug therapy due to their potentials to enhance and preserve the clinical therapeutic effects of chemo-drugs with less side effects by improving their protection, absorption, penetration and distribution 2, 4-6. Nanocarriers for drug delivery have several advantages 2, 7, 8: (1) protecting the drug from being degraded and prolonging the retention time in the body; (2) increasing the solubility of some hydrophobic drugs; (3) targeted delivery and controlled release of drugs by nanoparticles modification to keep the drug concentration in tumor sites and maximize therapeutic effects; (4) possibility of multiple drug delivery to achieve synergistic therapeutic response, or application of combination therapy such as chemo-photothermal therapy.

Osteosarcoma is the most common primary malignant bone tumor mainly occurred in childhood and adolescence 9. Due to the introduction of chemotherapy and advances in surgical technology, the 5-year survival rate of those with local osteosarcoma has improved to approximate 70% 9, 10. However, current chemo-drugs commonly used in treatment of osteosarcoma are limited for their side effects and development of resistance 11. To address these drawbacks and to increase the efficacy of chemotherapy, a variety of nanoplatforms for targeted drug or gene delivery has been extensively investigated in osteosarcoma, and nanotechnology has been proposed as a promising strategy for osteosarcoma treatment 7, 12-14.

In this review, we retrospectively summarized the recent advances of nanocarriers for targeted drug delivery in osteosarcoma. We discuss the commonly used types of drug delivery nanoparticles, controlled drug release upon different stimuli, and nanocarriers modification strategies for targeted drug delivery. The application of nanoparticles in the management of metastatic osteosarcoma is also briefly discussed. And we hope this review will provide readers a general understanding of current status in osteosarcoma nanomedicine, and inspire further investigations in novel drug delivery nanosystems for osteosarcoma treatment.

## Types of nanoparticles

Nanosized drug delivery systems can be roughly classified into organic and inorganic carriers 7. Organic nanocarriers reported for osteosarcoma drug delivery mainly include liposomes, polymers, micelles, and dendrimers. And inorganic nanocarriers mainly include metallic nanoparticles, mesoporous silica nanomaterials, carbon-based nanomaterials, and calcium phosphates carriers. However, it is difficult to obtain multifunctional and intelligent nanocarriers from single nanomaterial. Thus, current designed drug delivery nanosystems are usually nanocomposites of different kinds of materials.

### Organic nanocarriers

#### Liposomes

Liposomes are spherical vesicles with a hydrophilic cavity surrounded by one or several lipid bilayers that allows the encapsulation of drugs with different solubility. Hydrophobic drugs can be entrapped by the lipid bilayer and hydrophilic drugs can be encapsulated in the central aqueous core 3, 15, 16. Liposomal formulations are the first nanosized drug delivery carriers that have been successfully translated into clinical applications. And many liposomes for cancer therapy have been approved by the US Food and Drug Administration, or have underwent different clinical trials, including in osteosarcoma treatment 16-18. In addition to the inherent advantages such as biocompatibility and biodegradability, novel liposomes with different modification exhibit better selectivity, less systemic clearance, longer circulatory time, and controllable drug release 19.

A variety of nanoscale liposomes for anti-osteosarcoma agent delivery have been explored in the past years. Clinical trials have demonstrated that inclusion of liposomal muramyl tripeptide phosphatidyl ethanolamine (L-MTP-PE) could clinically and significantly improved the long-term survival of osteosarcoma patients 20. Normal liposomes can be recognized and cleared by the reticuloendothelial system (RES). Surface modification with biocompatible hydrophilic polymers, such as polyethylene glycol (PEG), could help liposomes to escape from RES and prolong the circulation time 17, 19. Recently, a PEGylated liposomal nanocarrier co-loaded with gemcitabine and clofazimine was reported and its anti-osteosarcoma effects were investigated 21. The hydrophilic gemcitabine was encapsulated in the aqueous core and hydrophobic clofazimine sequestered in lipid bilayer. And this co-loaded nanoscale formulation was stable and exhibited synergistic cytotoxicity on osteosarcoma cells *in vitro* 21. Liu Y et al 22 used PEGylated liposomes coated with gold nanoshell to deliver betulinic acid, which is a kind of hydrophobic natural anti-tumor drug. Other smart PEGylated liposomal formulations containing DOX, a commonly used chemo-drug, were also reported in osteosarcoma treatment 23, 24. There are still some drawbacks of PEGylation including disturbance of interaction between liposomes and tumor cells, and induction of anti-PEG IgM antibodies which is considered to be responsible for accelerated blood clearance (ABC) phenomenon after repeated injection of PEGylated liposomes 17. Thus, other polymers such as chitooligosaccharides (COS) have been investigated for liposome modification 25. This COS modified DOX-loaded liposomes showed good biocompatibility, prolonged circulation time, enhanced intracellular uptake, and improved anti-osteosarcoma effect. In addition to delivering anti-tumor drugs, liposomes are also appropriate vectors for gene delivery. PEGylated cationic liposomes are commonly used for siRNA loading and delivering, and could increase the stability of siRNA 26, 27.

#### Polymers

Many different polymers have been widely used for anti-cancer drug delivery and have received increased interest in recent years. Commonly used polymers from synthetic such as poly lactide-co-glycolic acid (PLGA) and PEG, or from natural origin such as hyaluronan and chitosan, demonstrate good biocompatibility and biodegradability. A range of biodegradable polymeric drug delivery systems designed for localized or systemic administration of therapeutic agents has been under clinical trials or approved for cancer treatment 28, 29.

Different biodegradable polymers have been used for designing safe and efficient nanocarriers for anti-osteosarcoma agent delivery. Suksiriworapong et al. 30 reported an easily synthesized methotrexate (MTX) conjugate with poly glycerol adipate (PGA). The MTX-PGA conjugates could self-assemble into nanoparticles that were physically and chemically stable but enzymatically degradable to release MTX. In another study, a natural polymer keratin nanoparticle functionalized with a photosensitizer Chlorin-e6, was prepared for Paclitaxel (PTX) loading 31. This natural biocompatible keratin, has exclusive tri-peptidic sequences, such as the “Arg-Gly-Asp” (RGD) and “Leu-Asp-Val” (LDV) sequences, that can specifically recognize vitronectin integrin receptors overexpressed in osteosarcoma cells 31. In addition to being used directly as drug delivery carriers, polymers are commonly used for nanocarriers modification to improve their stability, biocompatibility and specificity. For example, PEG-functionalized PLGA and polymer-lipid nanoparticles could prolong the systemic circulation time 32, 33. The natural polymer hyaluronic acid (HA) was an attractive ligand for targeted drug delivery to CD44-overexpressing tumors, and HA modification could enhance tumor cell internalization of these nanocarriers 34, 35. Due to its excellent biocompatibility and biodegradability, an *in situ* crosslinked nanogel based on HA has been synthesized for codelivery of DOX and cisplatin, two of the most widely clinically used chemo-drugs with proved synergistic effects, to osteosarcoma 36.

#### Micelles

Micelles are usually formed by amphiphilic polymers and have attracted considerable attention as promising nanocarriers for drug delivery. Polymeric micelles consist of a core and shell structure. In principle, the micelle core part is usually hydrophobic and can encapsulate poorly water-soluble agent, whereas the outer shell is able to stabilize the micelles in aqueous environment and can be modified with stimuli-responsive or tumor-targeting moieties 37-39. The size of these self-assembled micelles can be easily controlled by varying the length of the hydrophobic blocks. Compared with liposomes, micelles are considered to be more suitable for poorly water-soluble agents 39.

Several studies have reported different kinds of micelles for osteosarcoma treatment 40-42. Fang et al. 42 designed and synthesized an osteosarcoma targeted polymeric micelle carrier which was self-assembled from RGD-modified PEG-block-poly (trimethylene carbonate) (RGD-PEG-PTMC) amphiphilic block copolymers, for DOX delivery. Stewart A. Low et al. 40 designed a different DOX conjugate micellar delivery system for osteosarcoma therapy. In this study, the hydrophilic D-aspartic acid octapeptide was used as bone targeting agent and hydrophilic micelle corona; The DOX was loaded via an acid-sensitive hydrazone bond and served as the hydrophobic center to stabilize the micelle because of its hydrophobic nature as well as an ability to π-π stack with itself. The insertion of 11-aminoundecanoic acid (AUA) between DOX and the aspartic acid octapeptide could vary the hydrophobicity of this micelle-forming unimer 40. Another study reported that a polymeric micelle was synthesized to carry an arsenical drug, PENAO. The drug was chemically conjugated to the micelle surface to avoid drug leakage and premature release without altering PENAO's arsenous acid residue activity 41. Recently, an amphiphilic block copolymer PEG-poly[2-(methylacryloyl) ethylnicotinate] (PEG-PMAN) was prepared to deliver Zinc phthalocyanine (ZnPc), a poorly soluble photosensitizer for cancer photodynamic therapy (PDT). The formed polymeric micelles dramatically improved the solubility, blood circulation time and cell uptake of ZnPc, and exhibited excellent photodynamic therapeutic effects both *in vitro* and *in vivo* 43.

Even if micelles are highly stable in aqueous environment due to their low critical micellar concentration, they may also have a tendency to be dissociated in dilution or high ionic strength. A way to overcome this problem is introducing the cross-linking bridges in the hydrophobic core or in the hydrophilic shell, and thus regulating the drug release 38, 44.

#### Dendrimers

Dendrimers are nanoscale, globular, radially symmetric, water-soluble macromolecules with well-defined sizes, branched structures, and high density of modifiable functional groups 15, 45. Furthermore, the abundant tertiary amines in dendrimers facilitates the release of nucleic acid or drugs from endosomes through a “proton sponge” effect 45, 46. Due to the above properties, dendrimers are attractive nanocarriers for drug and gene delivery. Drugs can be either encapsulated in their internal core by noncovalent interactions or conjugated to their surface functionalities by covalent linkages 46. And cationic dendrimers are also ideal nanocarriers for gene delivery because the abundant cationic groups not only provide a variety of nucleic acid attaching sites but also increase the gene transfection efficiency 46-48. However, highly positively charged dendrimers could strongly interact with the negatively charged cell membranes and cause cytoplasmic contents leakage and subsequent lysis, which raises concern regarding their safety 46, 49. Surface modification is a commonly used strategy to reduce the charge and overcome these drawbacks.

Dendrimers has been investigated as chemo-drug or gene delivery systems in osteosarcoma. Recently, a new type of nanogels containing DOX was synthesized by incorporating generation 5 (G5) PAMAM dendrimers and DOX into alginate (AG) nanogels 50. The presence of G5 dendrimers improved the stability, DOX loading capacity and drug release sustainability of the nanogels. Meanwhile, coating of AG could shield the charge of the dendrimers and improved the biocompatibility of the dendrimers Furthermore, the researchers found the DOX-loaded nanogels could be effectively internalized by human osteosarcoma cells and intracellularly delivered DOX to exert its cytotoxicity 50. Dendrimers were also investigated as gene delivery vectors in osteosarcoma. A triazine-modified dendrimer G5-DAT66 has been prepared and used for TRAIL gene delivery 51. This modified gene vector showed good water solubility and more superior transfection efficiency than commercially transfection reagents such as Lipofectamine 2000 and SuperFect, and significantly inhibited the osteosarcoma growth *in vitro* and *in vivo*.

### Inoganic nanocarriers

#### Metallic nanocarriers

Metallic nanocarriers can be pure metallic particles such as gold, silver, and copper; or metallic compound such as oxides and Mxene; or hybrid polymers that consist of metal ions or clusters such as metal organic frameworks (MOFs) 15, 52, 53.

Among the pure metallic nanoparticles, gold and silver nanoparticles are the most commonly investigated in osteosarcoma therapeutics. Due to the remarkable properties such as high surface area to volume ratio, stable nature, multi-functionalization, facile synthesis, high permeability and retention effect, and photothermal conversion capability, gold nanoparticles (AuNPs) have long been considered as a potential tool for cancer treatment 54, 55. A study from Rahim et al. showed that spherical glycogenic AuNPs could inhibit the growth of osteosarcoma cell 56. Steckiewicz et al. 57 assessed the effect of AuNPs shape on their cytotoxicity against osteosarcoma cells and demonstrated that the AuNPs stars were more cytotoxic than rods and spheres. AuNPs as drug or gene delivery carriers were also reported in osteosarcoma 58-60. Gold nanoshells were reported to have strong absorption in near infrared (NIR) region and high photothermal conductivity. Liu Y et al. designed a gold nanoshell-coated liposomal drug delivery system 22. Upon NIR irradiation, the nanocarriers could rapidly transform NIR light to heat, increase cellular uptake, and trigger the release of drug. In addition to AuNPs, the cytotoxic effect of silver nanoparticles (AgNPs) were also investigated in osteosarcoma 61,62. Reactive oxygen species (ROS) generation leading to mitochondria-dependent apoptosis was considered as the possible mechanisms of AgNP-induced cytotoxicity.

Metallic compound-based nanoparticles reported in osteosarcoma treatment are mainly metallic oxides, and these metallic oxide nanoparticles can serve as intrinsic therapeutic agents without the need of loading chemotherapy drug. For example, the anti-cancer effect of titanium dioxide (TiO2), terbium oxide (Tb2O3), zinc oxide (ZnO) and cerium oxide (CeO2) nanoparticles has been evaluated verified in osteosarcoma cells 63-65. However, the biocompatibility and antitumor effect *in vivo* were not further explored in these studies. Among the metallic oxide nanoparticles, iron oxide such as ferroferric oxide (Fe3O4) was the most commonly investigated nanomaterials in osteosarcoma. And these nanoparticles were mostly used for thermal therapy due to its ability to convert the energy of magnetic field into heat 66-68. Besides, iron oxide nanoparticles could also be used for drug delivery because of its biocompatibility. Popescu et al. successfully fabricated Gemcitabine conjugated Fe3O4 nanoparticles. And this nanoconjugate showed promising results regarding their cytotoxicity against human osteosarcoma cells 69. The superparamagnetic properties of iron oxide could increase the cellular uptake of loaded cargos under an external magnetic field 70. However, Fe3O4 nanoparticles were reported to have a tendency to agglomerate in biological conditions 68. Therefore, it is necessary to modify the Fe3O4 nanoparticles' surface to overcome the problem when used for different biomedical applications. Other metallic nanomaterials mentioned above (Mxene and MOFs) as drug delivery systems have not been reported in osteosarcoma treatment.

#### Mesoporous silica nanocarriers

Mesoporous silica nanoparticles (MSNs) have attracted considerable attention for drug or gene delivery because of their excellent characteristics including simple fabrication process, uniform morphology, variable particle size, modifiable surface, tunable pore size and volume, and FDA recognized biosafety 71, 72. The large surface area and the porous structure enable MSNs to have high loading capacity with different agents. Surface modification with different functional groups allows MSNs to realize tumor targeting and controlled drug release 72.

The use of MSNs as drug or gene delivery systems in osteosarcoma have also been widely reported. Shahabi et al. 73 evaluated the influence of MSNs surface modification on the encapsulation and release of DOX, as well as cancer cell response in the absence or presence of serum proteins. They demonstrated that, in the presence of serum proteins, sulfonate functionalization of MSNs showed both increased doxorubicin loading and *in vitro* doxorubicin delivery rate, compared with unfunctionalized MSNs, antibody-conjugated MSNs or even free DOX. Hartono and colleagues 70 designed a new type of PEI modified and iron oxide loaded large pore MSNs for gene delivery to osteosarcoma cells. The magnetic property of iron oxide promotes the cellular uptake of MSNs under an external magnetic field. PEI covalently linked on the MSNs improves the particle's affinity against siRNA and cells membrane which can also increase cell uptake. The 'proton sponge effect' of PEI enables the particle to effectively deliver the siRNA and escape from endosome, thus increasing the transfection and silencing effects. Another research also fabricated a similar type of magnetic core-shell silica nanoparticles to delivery siRNA 74. An additional acid-liable coating with tannic acid could further protect the siRNA and serve as a pH-responsive releasing switch. Paris et al. 75 reported a smart hierarchical ultrasound-responsive MSN for drug delivery. The PEG shell will be detached from the MSNs by ultrasound-induced temperature increase, exposing the positively charged surface, which favors the cell internalization of the particles and enhance the cytotoxic effect. Martínez-Carmona et al. 76 developed a tumor-targeted and PH-responsive MSNs loaded with DOX for osteosarcoma treatment. This nanoscale drug carrier could improve the antitumor effectiveness and decrease the toxicity to normal cells. Studies from Lu et al. 77 demonstrated that functionalized smart MSNs showed a high specificity for osteosarcoma, and exhibited significantly synergistic photothermal-chemotherapeutic properties.

Because of the superior nature such as safety, high drug loading capacity, controllable drug release and modifiable surface, MSNs are considered to have the potential to be a more promising platform for cancer therapy compared to other inorganic nanocarriers such as copper, gold, and silver which exhibit some cytotoxicity. However, there is still a long approach before clinical translation and commercialization 71, 72.

#### Carbon-based nanocarriers

Carbon-based nanomaterials such as carbon nanotubes (CNTs), graphene oxide (GO), mesoporous carbon, and carbon dots, have drawn considerable attention and been extensively investigated for cancer therapy because of their good physicochemical properties including easily-modified surface, exellent photo-thermal conversion ability, supramolecular π-π stacking, and high adsorption ability 78-82.

Among these different carbon nanomaterials, GO and CNTs were the mostly reported in osteosarcoma. Tang et al. 83 evaluated the toxicity and underlying mechanisms of GO on osteoasrcoma cells in the absence of fetal bovine serum which excluded the formation of the blood protein-graphene corona and enabled the direct interaction of GO with the cell membrane, and they found that different mechanisms including ROS generation, apoptosis, and autophagy were involved in GO-induced anti-osteosarcoma effect. Another study 84 described the metabolomic response of osteosarcoma cells to GO-mediated hyperthermia. Upon NIR irradiation, the levels of glutamate and uridine nucleotides decreased, and glycerophosphocholine increased, which may reflect laser-induced membrane damage. Recently, a PH-sensitive graphene oxide-chitosan nanoparticle was developed to carry siRNA, and this nanocarrier exhibited effective release of siRNA in acidic condition 85. Li et al. 86 reported that anti-HER2 antibody trastuzumab (TRA) was noncovalently conjugated to GO to form stable TRA /GO nano-complexes. And this TRA /GO nanoscale formulation demonstrated significantly enhanced HER2-binding activity and effective anti-osteosarcoma capacity.

CNTs have also attracted considerable attention for cancer therapy. Yan et al. 87 constructed a 3D structured graphene/single‐walled carbon nanotubes (G/SWCNT) hybrid by combining single-walled carbon nanotubes and graphene, and evaluated its cytotoxicity of on osteosarcoma cells. The G/SWCNT hybrids showed less cytotoxic than graphene and SWCNTs, and the G/SWCNT hybrids-induced apoptosis was through ROS-mediated mitochondrial pathway. Polymers were commonly used to modify CNTs to reduce the cytotoxicity 88, 89. Zhang et al. 89 fabricated a polyamidoamine (PAMAM) dendrimers functionalized multi-walled carbon nanotubes (MWCNTs). The hybrids exhibited good dispersibility and stability in aqueous solution, excellent biomolecule immobilization ability, and reduced cytotoxicity. PLGA modified CNTs were developed by Cheng et al. 88 for delivering pro-apoptotic protein caspase-3. This conjugate showed a high transfection rate and significant anti-osteosarcoma effect *in vitro*. Another research demonstrated that SWCNTs could specifically inhibit the process of TGFβ1-induced osteosarcoma cells dedifferentiation, prevent the stem cell phenotypes acquisition and reduce the osteosarcoma stem cells viability 90.

Though many exciting advances in the field of carbon-based nanomaterials have been made, yet there were relatively few investigations of these nanomaterials as drug delivery systems in osteosarcoma. More focused and systematic research on tumor targeting, toxicity and pharmacokinetics of these nanomaterials is needed in osteosarcoma.

#### Calcium phosphates nanocarriers

Calcium phosphates (CaP) nanoparticles, particularly hydroxyapatite nanoparticles (HANPs), are considered as promising nanocarriers to bone tissues because they are biocompatible, biodegradable, non-immunogenic, PH-sensitive and facilely modifiable, and have shown to be preferentially accumulated in bone tissues 91-94. CaP-based nanoparticles have been widely used for delivery of anticancer drugs in osteosarcoma treatment 92, 93, 95-97.

Son et al. 93 fabricated a series of CaP-alginate nanocomposites loading different anticancer drugs. The CaP-polymer-drug complexes were formed by hydrogen bonding and electrostatic interaction. These drug-loaded nanocomposites showed a prolonged drug release at PH 7.4, and faster release at PH 4.5, and exhibited anticancer activity on osteosarcoma cells. Based on the idea that like should cure like and that bone diseases and deformities are best targeted and treated using one or more components of bone itself, hydroxyapatite, a mineral matrix that resembles the crystallographic structure of natural bone, has been considered as an ideal carrier for drug delivery to bone diseases, such as osteosarcoma 92, 95, 97. In addition, HANPs have been reported to possess the ability for inhibiting cancer cell growth *in vitro* and *in vivo*, and show less cytotoxicity to normal cells 98. Wu et al. 97 modified HANPs with bisphosphonate as a bone targeting moiety to deliver anticancer drug JQ1, a small-molecule bromodomain inhibitor. Loading JQ1 onto HANPs can delay its release and prolong the retention in the body. Though the JQ1-loaded HANPs exhibited a promising selectivity, being more toxic to osteosarcoma cells than to primary fibroblasts, the comparatively low loading efficiency of hydrophobic JQ1 onto ionic HANPs has been a drawback of this therapy. Wang et al. 96 developed a novel kind of biodegradable and pH-sensitive selenium-doped hydroxyapatite nanoparticles (Se-HANs), and evaluated their anti-osteosarcoma effects and mechanisms *in vitro* and *in vivo*. The selenium released from Se-HANs could induce tumor cell apoptosis through an inherent caspase-dependent apoptosis pathway synergistically orchestrated with ROS generation.

## Strategies for osteosarcoma-targeted drug delivery

**Figure 1:**
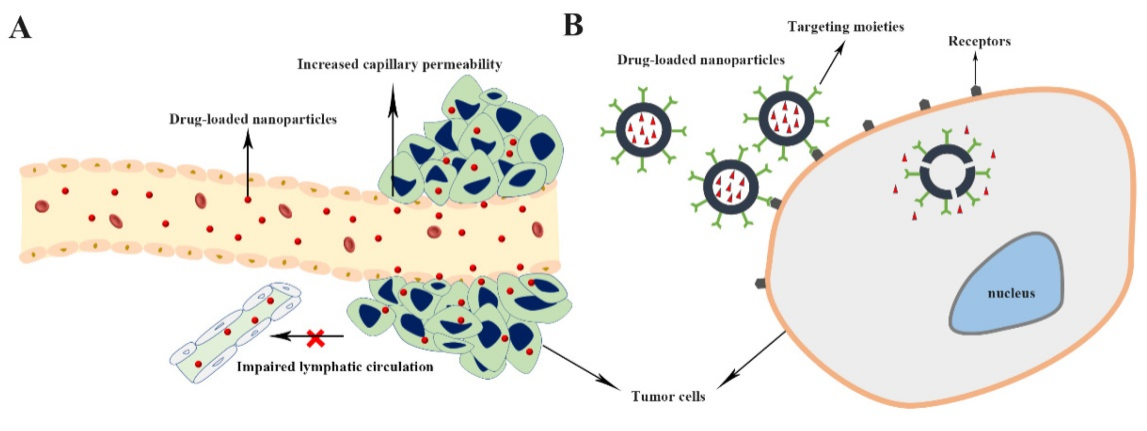
A simple schematic illustration of passive (A) and active (B) tumor targeting mechanism of drug delivery nanodevice.

Targeted drug delivery generally means delivery of the intravenously administered drugs to the target site, e.g. tumors. Targeted drug delivery systems are designed to facilitate drug delivery to the tumor sites with minimum side effects, and that are performed by two targeting strategies, including passive and active targeting 99 (Figure 1). Compared with free small therapeutic agents, nanocarriers can passively accumulate in tumors through the enhanced permeability and retention (EPR) effect, which is characterized by leaky blood vessels and impaired lymphatic drainage in tumor tissues, and achieve superior therapeutic efficacy, while reducing side effects 100, 101.

Even though nanocarriers can be passively targeted to tumor via EPR effect, it suffers from some serious limitations such as inefficient drug diffusion into tumor cells, the random nature of targeting, and the lack of EPR effect in some tumors. Thus, there is potential to improve the tumor targetability of the nanocarriers through active targeting strategies, such as ligand-mediated tumor targeting 8, 102, 103. Ligand-functionalized nanocarriers could interact with cancer cells and be internalized via receptor-mediated endocytosis mechanism, thereby resulting in higher therapeutic effect 102, 104. The knowledge of tumor cell epitopes and advances in nanotechnology have allowed the development of targeted nanocarriers able to actively deliver antitumor agents to diseased area 4, 8. Bisphosphonates 34, 97, aptamers 14, hyaluronic acid (HA) 24, folate 105, and peptides 42 have been reported to be used in designing osteosarcoma-targeted drug delivery systems (Table 1).

Bisphosphonates display a pyrophosphate-like structure and exhibit affinity toward bone by chelating with divalent calcium ions (Ca2+) present in the HAP matrix of the bone 4, 97, 106. Recently, HAP nanoparticles functionalized with medronate (the smallest bisphosphonate) as a bone-targeting moiety have been reported in osteosarcoma-targeted treatment 97. Though lacking animal studies, *In vitro* models showed that the JQ1-loaded HANPs significantly inhibited osteosarcoma cells migration and invasion but exhibited less cytotoxicity to primary fibroblasts 97. In addition to this, bisphosphonate-conjugated polymeric nanocarriers have been fabricated to deliver chemotherapeutic drugs to osteosarcoma, and the tumor-targeting ability and anticancer effects were evaluated *In vivo*. These functionalized, DOX-loaded nanoparticles demonstrated enhanced, prolonged tumor accumulation and significantly improved anticancer activities compared to nontargeted DOX-loaded nanocarriers or free DOX 107-109. However, bisphosphonates are bone-targeted rather than specifically osteosarcoma-targeted, and the prolonged residence in the bone tissue may have the potency to inhibit osteoclasts and bone homeostasis 106, 110.

Aptamers are short, synthetic and single-stranded DNA or RNA molecules that can specifically bind to their targets with a high affinity. And aptamers have attracted broad interest in targeted drug delivery because of their high selectivity and affinity, low immunogenicity, easy synthesis with high reproducibility, facile modification, and relatively rapid tissue penetration with no toxicity 111, 112. Liang et al. 113 used mouse OS cells (K7M2) as target cells to screen aptamers by cell-SELEX. Mouse normal hepatocytes (AML12) and peripheral blood mononuclear cells (PBMCs) were selected as negative cells for decreasing non-specific liver and PBMCs uptake after *In vivo* administration. Finally, LC09 were chose as osteosarcoma cell-targeted aptamers, and LC09-modified lipopolymers loading CRISPR/Cas9 plasmids encoding VEGFA gRNA and Cas9 were developed. The LC09-fuctionalized nanocomposites achieved selective distribution of CRISPR/Cas9 in both orthotopic osteosarcoma and lung metastasis, and reduced VEGFA expression and secretion, thus inhibiting osteosarcoma malignancy and lung metastasis. CD133 is considered to be a cancer stem cells (CSCs) marker in osteosarcoma or other tumors. Accordingly, CD133 aptamers have been used as targeting ligands for tracking osteosarcoma CSCs 14, 32, 114. CD133 aptamers-functionalized polymeric nanoparticles could specifically and efficiently deliver anticancer drugs to CD133 positive osteosarcoma CSCs, and significantly improve therapeutic effects than free drugs and non-targeted nanoparticles 32, 114. Moreover, epidermal growth factor receptor (EGFR) aptamers were also applicated in developing osteosarcoma-targeted drug delivery carriers 14, 115.

HA, an endogenous polysaccharide, has hydroxyl and carboxylic groups, as well as an N-acetyl group, which can be used for further chemical modifications. HA exhibits some superior physiochemical natures, such as biodegradability, biocompatibility, and non-immunogenicity 34, 116. Many cancer cells, including osteosarcoma, are known to overexpress HA-binding receptors, such as CD44 34, 116, 117. Chi et al. 24 developed a redox-sensitive, HA functionalized liposomal nanocarrier to improve chemotherapy of osteosarcoma. The HA-modified liposomes demonstrated a preferential internalization into MG63 cells over normal human hepatic cells. Furthermore, the strong cellular uptake of the HA-functionalized nanoparticles by MG63 was inhibited with pre-treatment with free HA due to the competitive binding with CD44 receptors. HA-modified nanoparticles showed a persistent selective tumor accumulation, and better tumor suppressive effects compared with non-HA coated nanoparticles. Recently, researchers from the same group developed a bone- and CD44-targeted liposomal drug delivery system by conjugating alendronate and HA as targeting moieties, respectively, to improve the osteosarcoma-targeting ability and specific intracellular drug delivery 34.

Folate is an ideal candidate for ligand-based targeted therapy, as numerous examples of folate receptor-targeted drug delivery carriers have been reported to transport anticancer drugs into cells through receptor mediated endocytosis 118, 119. The overexpression of the folate receptor has been reported in many osteosarcoma xenograft samples 120. Thus, folate-functionalized nanocarriers have been used in osteosarcoma-targeted therapy 103, 105, 121. Wang et al. 121 prepared a novel folate-functionalized nanoscale polysaccharide derivative as gene carrier, and assessed the safety and anti-osteosarcoma effects of this nanocomplex. Folate modification improved cellular uptake of the complexes into osteosarcoma cells, and cellular uptake decreased with free folate competition. The folate conjugated nanocomplexes demonstrated a better antitumor effect *in vitro* and *in vivo* than the nontargeted complexes.

In addition, peptide ligands, such as RGD and YSA, have shown active osteosarcoma cell targeting ability 42, 77, 122. RGD, a cell-affinitive peptide, is able to interact with αvβ3 and αvβ5integrins, which are widely expressed in osteosarcoma cell lines. Studies from Fang et al. 42 demonstrated the RGD-modified polymeric micelles exhibited enhanced cell uptake compared to the nontargeted counterparts, displaying specific osteosarcoma cells targeting and killing ability *In vitro* over healthy osteoblast cells. YSA, a 12-amino acid peptide, is a ligand for ephrin type-A receptor 2 (EphA2), a surface molecule which is overexpressed in osteosarcoma cells and tissues 123. DOX-loaded liposomes modified with YSA peptide could efficiently target human Saos2 osteosarcoma cells, and increase toxicity and cellular uptake 122.

Osteosarcoma is characterized as high-grade malignant tumor due to the high local aggressiveness and rapid systemic metastasis, especially lung metastasis, and the 5-year survival rate is only about 15-30% for patients with lung metastasis. 121, 124. Thus, improving the prognosis of metastatic osteosarcoma remains one of the main challenges for clinicians and researchers. Nanoscale drug delivery systems can be promising strategies in the management of metastatic tumors. First, drug-loaded nanoparticles can be actively or passively delivered to primary and metastatic tumor sites. Second, well designed nanocarriers can be conjugated with certain targeting moieties, such as ligands of cell adhesion molecules which are usually overexpressed on the surface of invasive tumor cells, to combat these aggressive cells; Also, drug-loaded nanocomposites can influence the function of invasive cells to exert anti-metastatic effects. Moreover, functional drug delivery system can also re-educate the tumor environment which involves tumor cells, immune cells, stomal cell, etc., to block the initiation of metastasis 125-128. Recently, edelfosine (ET), a potential anti-tumor agent with poor oral bioavailability and severe side effects, was encapsulated in lipid nanoparticles (ET-LNs) for osteosarcoma treatment by Gonzalez-Fernandez et al. 126. Results of this study demonstrate that orally administered ET-LNs show significant growth inhibition effects in primary osteosarcoma cells. Besides, this drug-loaded nanosystem exhibits outstanding anti-metastatic potential as reflected by less lung metastatic nodules in mice receiving ET-LNs. Su et al. 127 designed a PEG functionalized redox-responsive dimeric paclitaxel (diPTX)-loaded cationic polycarbonates micelle (diPTX@CPGC) and evaluated the anti-tumor effects towards lung metastases via aerosol-based administration. The innovative nanocomposites exhibit well penetration capacity and significantly suppress the progression of lung metastases with minimal side effects. In another study, the effects of bovine serum albumin-Zinc phthalocyanine (BZ)-induced photodynamic therapy (PDT) on osteosarcoma were investigated 128. Surgery together with PDT was applied in the orthotopic xenograft model, and tumor growth and recurrence were significantly inhibited. Moreover, BA-induced PDT, especially combined with autophagy inhibitor, could downregulated the expression of PD-L1 and activate the immune response, thus inhibited tumor metastasis.

## Controlled release of loaded drugs

Precise and selective drug release in the tumor sites will further enhance the therapeutic efficacy and minimize the undesired side effects to normal tissues 5. To this end, different kinds of stimuli-responsive nanocarriers have been developed. With well-designed chemical composition or physical structure, these nanoscale drug delivery systems can be triggered by either intrinsic (e.g. pH, redox, and enzyme) or external (e.g. light, magnetic field, and ultrasound) stimuli, thus providing spatiotemporally controllable drug release to potentiate the anti-cancer efficacy 5, 100, 129. Here, we simply describe the application of some of these stimuli-responsive nanosystems in osteosarcoma (examples were listed in Table 2).

### pH-responsive nanocarriers

The pH of blood is approximately 7.4, whereas extracellular pH in tumors decreases to 6.0-7.2. The pH in the subcellular compartments decreases to 5.0-6.0 in endosomes and 4.0-5.0 in lysosomes 100. Through the introduction of acid-sensitive linkers, such as acetal, hydrazone, and glycerol ester groups, these pH-sensitive nanocarriers could store and stabilize antitumor drugs at physiological pH, but rapidly release the drugs at an acidic environment 100, 130, 131. In a recent study, mesoporous zinc hydroxyapatite (ZnHAP) decorated with pluronic block copolymer, F127, was synthesized and used as a carrier for drug delivery 132. The standard chemotherapeutic drug methotrexate (MTX) was grafted onto the surface of the nanoparticles via amide bond. In order to simulate the cytosol and the endosomal/lysosomal conditions, the release of MTX from the nanoparticles was evaluated under different pH values ranging from 4 to 7.4 in the presence of crude protease from bovine pancreas, and a large amount of MTX release was observed at pH 4.0 132. Another research group has designed a multifunctional nanodevice acting as drug delivery platforms with pH-sensitivity 76. A polyacrylic acid (PAA) shell was anchored to the MSNs surface via an acid-cleavable acetal linker, preventing premature drug release and providing the nanocarrier of pH-responsive capability. The drug release rate at PH 5.3 was much higher than PH 7.4 in both protein-free phosphate buffered saline (PBS) and protein-containing cell culture medium. Chitosan have been used to functionalize drug carriers, enabling the composite carriers to possess pH-responsive performances and show high drug release rate in the slightly acidic environment than in the neutral environment 131.

### Redox-responsive nanocarriers

Glutathione (GSH), a highly effective antioxidant, can reduce the disulfide bonds of nanocarriers. GSH present in intracellular environments (2-10 mM) is 100-fold higher than that in extracellular environments (2-10 μM). The concentration of GSH in the cancer cells was found to be much higher than that in normal cells. The difference in the redox potential between intracellular and extracellular concentration of GSH can be used for intracellularly controlled drug release once the nanocarrier is internalized 100, 133. Chi et al. 24 developed redox-sensitive and tumor-targeted nanocarriers to improve chemotherapy of osteosarcoma. The liposomes were stabilized with a novel detachable PEG conjugated with cholesterol through a reducible disulfide linker. *In vitro* release of DOX was well controlled at physiological conditions, but a burst release of more than 60% was observed in the presence of 10 mM GSH, compared to non-redox sensitive nanocarriers.

### Light-responsive nanocarriers

Due to its noninvasiveness and spatiotemporal precision, light with a specific wavelength has been extensively used as an external stimulus for triggering on-demand drug delivery 129. Recently, a visible light-responsive drug delivery MSN was designed, and drug release and antitumor activity of this smart nanocarrier were evaluated in osteosarcoma cells 134. The pore outlets of drug-loaded MSN were blocked with porphyrin nanocaps through ROS-cleavable linkages. Upon visible light irradiation, porphyrin nanocaps could generate ROS species that are able to break the sensitive bonds and therefore triggering pore uncapping and allowing drug release. However, limited tissue penetrating capability of this short-wavelength light is the main drawback for *In vitro* and *In vivo* experiments. Near infrared (NIR) light has received considerable interest in photoactivated drug delivery because of its unique advantages such as deep tissue penetration, and limited photo damage 135. In addition, some nanomaterials which have strong light absorption capacity in the NIR regions could convert photo energy into heat, increasing localized temperature which will trigger the drug release from nanoplatforms 135. So far, different drug delivery nanocarriers based on NIR light absorbing nanomaterials have been reported in osteosarcoma treatment, and exhibited good NIR-responsive drug release ability 77, 121. Lu et al. 77 developed a new type of mesoporous silica-coated bismuth sulfide nanoparticles (Bi2S3@MSN) encapsulating DOX. Researchers found that the drug release efficiency of the Bi2S3@MSN upon NIR light irradiation was significantly improved, even with an ultralow power density (0.5 W/cm2). The drug loaded nanoparticle combined with NIR irradiation could significantly ablate the tumors, leading to efficient suppression of the malignant sarcoma.

### Magnetic field-responsive nanocarriers

Magnetic nanoparticles (e.g. iron oxide nanoparticles) can convert magnetic energy into heat when exposed to an alternating magnetic field. The heat generated by these particles may cause structural alteration of the drug loaded nanocarriers, thus achieving an “on demand” drug release 136, 137. The application of magnetic field-responsive nanomaterials for drug delivery was rarely reported in osteosarcoma. Jalili et al. 138 developed an injectable nanoengineered hydrogel by combining thermo-responsive polymers and magnetic nanoparticles for localized and on-demand delivery of DOX. The drug release of this nanocomposite was temperature responsive, and altering magnetic fields triggered much more drug release. However, *in vivo* biosafety, biodistribution, drug release kinetics, and antitumor effects of this drug-loaded nanogel were not evaluated in this study.

## Conclusions and future perspectives

The unknown etiology, high genetic instability, large histological heterogeneity, lack of specific biomarkers, high local aggressiveness, and a rapid metastasizing potential create challenges for osteosarcoma treatment 124. Despite the efficacy of chemo-drugs on osteosarcoma, there are still some drawbacks such as toxicity to normal tissues, development of drug resistance, and rapid blood clearance 4,124. Thus, various nanoplatforms capable of delivering the therapeutic agent rightly to the tumor site have been developed to improve the therapeutic effects and minimize side effects. In this review, we discussed different nanocarriers that are commonly used as emerging tools for the treatment of osteosarcoma. Although exciting progressions in the understanding of tumor biology and development of various multifunctional drug delivery platforms may offer great promise for osteosarcoma treatment in the future, these nanomaterials are not well-developed for use in osteosarcoma patients for the present. Most of them are still at the cellular and animal experimental stage, and there is a long transitional period before clinical application 110. More optimized nanocarriers for demanded drug delivery will be obtained after overcoming some challenges 78, 107, for example, 1) An ideal drug delivery system should selectively accumulate in the tumor sites, thus deeper understanding of the osteosarcoma targeting mechanism should be further explored for finding more specific target ligands; 2) Accumulation of nanocarriers in the liver is a common challenge for all drug delivery nanoplatforms; 3) Light currently used for photothermal therapy has a limited penetration ability towards deep tumors, therefore other alternative strategies should be developed; 4) Long-term biosafety of some of these nanomaterials should be systematically evaluated using more relevant animal models *In vivo*, and furthermore, animal models are different from human tissue, after all.

## Figures and Tables

**Table 1:** Examples of osteosarcoma-targeted strategies used in drug delivery nanomaterials and the therapeutic potential

NPs Types	Targets	Cargos	Tested models	Effects	Ref.
HANPs	Medronate	JQ1	***In vitro***: K7M2 murine OS cells, and mouse primary lung fibroblasts ***In vivo***: none	***In vitro***: selectivity, and more toxic to OS cells than to primary fibroblasts ***In vivo***: none	^97^
LbL liposomes	alendronate	DOX	***In vitro***: 143B OS cells; ***In vivo***: 143B xenografts in nude mice	***In vitro***: rapid and effective cell uptake ***In vivo***: preferential accumulation in the xenografts, enhanced chemotherapeutic effects in tumor-bearing mice, extended animal survival	^107^
BP NPs	BP	DOX	***In vitro***: Saos-2 OS cells; ***In vivo***: Saos-2 subcutaneous xenograft tumor in a nude mouse model	***In vitro***: higher cellular uptake and therapeutic effect than free DOX ***In vivo***: tumor targeting ability, prolonged retention in tumor site, enhanced bone tumor toxicity	^108^
LipopolymerNPs	LC09 aptamers	CRISPR/Cas9 plasmidsencoding VEGFA gRNA and Cas9	***In vitro***: K7M2 and K12 mouse OS cells ***In vivo*:** BALB/c nude mice bearing K7M2 orthotopic xenograft	***In vitro***: enhanced cellular uptake, effective gene silencing, improved antitumor effects ***In vivo***: tumor cell-selective distribution and expression of CRISPR/Cas9, superior tumor suppression effect	^113^
polymeric NPs	CD133 aptamers	salinomycin	***In vitro***: Saos-2 CD133^+^ and CD133^-^ OS cells ***In vivo***: Saos-2 subcutaneous xenograft tumor in a nude mouse model	***In vitro***: specifically internalized by CD133^+^ OS cells, enhanced cytotoxic effect to CD133^+^ OS cells ***In vivo***: superior antitumor activity, decreased CD133^+^ OS cells proportion	^32^
lipid-polymer NPs	CD133 aptamers	ATRA	***In vitro***: Saos-2 (U-2OS) CD133^+^ and CD133^-^ OS cells ***In vivo***: BALB/c nude mice bearing Saos-2 subcutaneous xenograft	***In vitro***: specifically targeting to CD133^+^ OS cells, enhanced cytotoxic effect to CD133^+^ OS cells ***In vivo***: enhanced antitumor activity, decreased CD133^+^ OS cells proportion	^114^
polymer-lipid hybrid NPs	EGFR aptamers	salinomycin	***In vitro***: U-2OS and MG63 OS cells ***In vivo***: none	***In vitro*:** increased cellular uptake and cytotoxic effect compared with non-targeted NPs and free salinomycin, decreased CD133^+^ OS cells proportion ***In vivo*:** none	^115^
liposomes	HA	DOX	***In vitro***: MG63 OS cells and Normal human hepatic LO2 cells ***In vivo***: MG63 xenograft mouse model	***In vitro***: preferentially internalized to MG63 over LO2 cells, higher cytotoxicity to MG63 cells compared with non-HA coated liposomes ***In vivo***: strong and persistent selective tumor accumulation, enhanced antitumor effects	^24^
liposomes	HA and alendronate	DOX	***In vitro***: MG63 cells ***In vivo***: BALB/c nude mice bearing MG63 orthotopic xenograft	***In vitro***: rapid internalization, dual targeting liposomes were more toxic than other liposomes but less toxic than free DOX ***In vivo***: enhanced tumor targeting ability and antitumor effects	^34^
polysaccharide derivative NPs	folate	AEG-1 siRNA	***In vitro***: 143B and U-2OS cells ***In vivo***: 143B cells tumor-bearing mice models	***In vitro***: enhanced cellular uptake and transfection efficiency, increased anti-proliferation and anti-invasion ability ***In vivo***: enhanced tumor suppressive effects compared to non-targeted nanocomplex	^121^
polymeric micelle	RGD	DOX	***In vitro***: MG63 and MNNG/HOS cells ***In vivo***: none	***In vitro***: enhanced cell targeting ability and more effective anti-tumor effect ***In vivo***: none	^42^
MSNs	RGD	DOX	***In vitro***: rat UMR-106 OS cells ***In vivo***: UMR-106 cells tumor-bearing mice models	***In vitro***: enhanced tumor cell uptake ***In vivo***: outstanding tumor targeting ability	^77^
liposomes	YSA	DOX	***In vitro***: Saos-2 OS cells ***In vivo***: none	***In vitro***: higher and more nuclear uptake than non-targeted liposomes ***In vivo***: none	^122^

NPs, nanoparticles; HANPs, hydroxyapatite nanoparticles; JQ1, a small-molecule bromodomain inhibitor; OS, osteosarcoma; LbL, layer-by-layer; DOX, doxorubicin; BP, bisphosphonate; ATRA, all-trans retinoic acid; EGFR, epidermal growth factor receptor; HA, hyaluronic acid; AEG-1, astrocyte elevated gene-1; RGD, arginine-glycine-aspartic acid peptide; MSNs, mesoporous silica nanoparticles; YSA, a 12- amino acid peptide which is an Ephrin A1 mimic and a ligand for EphA2.

**Table 2:** Examples of stimuli-responsive nanomaterials reported in osteosarcoma

Nanomaterials	Stimuli	Cargos	Ref.
PAA-MSNs	pH	DOX	76
F127@ZnHAP	pH	MTX	132
ZSM-5 /CS NDs	pH	DOX	131
liposomes	redox	DOX	24, 25, 34
Bi_2_S_3_@MSNs	NIR/temperature	DOX	77
MSNs	visible light	TOP	134
GelMA/(poly(NIPAM-co-AM)/MNPs) nanogels	magnetic field /temperature	DOX	138

PAA, polyacrylic acid; F127@ZnHAP, mesoporous zinc-substituted hydroxyapatite nanoparticles were decorated with F127 (pluronic block copolymer); MTX, methotrexate; ZSM-5 /CS NDs, mesoporous ZSM-5 zeolites/chitosan core-shell nanodisks; TOP, topotecan; GelMA, gelatin methacrylate; poly(NIPAM-co-AM), poly(N-isopropylacrylamide-co-acrylamide); MNPs, magnetic nanoparticles.
